# Anterior Corpectomy and Plating with Carbon-PEEK Instrumentation for Cervical Spinal Metastases: Clinical and Radiological Outcomes

**DOI:** 10.3390/jcm10245910

**Published:** 2021-12-16

**Authors:** Sokol Trungu, Luca Ricciardi, Stefano Forcato, Antonio Scollato, Giuseppe Minniti, Massimo Miscusi, Antonino Raco

**Affiliations:** 1 Neurosurgery Unit, Cardinale G. Panico Hospital, 73039 Tricase, Italy; stefano.forcato@gmail.com (S.F.); ascollat@hotmail.com (A.S.); 2 NESMOS Department, Sant’Andrea Hospital, Sapienza University of Rome, 00189 Rome, Italy; ricciardi.lu@gmail.com (L.R.); massimo.miscusi@gmail.com (M.M.); antonino.raco@gmail.com (A.R.); 3 Radiation Oncology Unit, Department of Medicine, Surgery and Neurosciences, University of Siena, 53100 Siena, Italy; giuseppeminniti@libero.it; 4 IRCCS Neuromed, 86077 Pozzilli (IS), Italy

**Keywords:** cervical spinal metastasis, carbon-PEEK implants, anterior cervical corpectomy, vertebral metastasis, minimally invasive surgery

## Abstract

**Background**: Anterior cervical corpectomy and plating has been recognized as a valuable approach for the surgical treatment of cervical spinal metastases. This study aimed to report the surgical, clinical and radiological outcomes of anterior carbon-PEEK instrumentations for cervical spinal metastases. **Methods**: Demographical, clinical, surgical and radiological data were collected from 2017 to 2020. The Neck Disability Index (NDI) questionnaire for neck pain, EORTC QLQ-C30 questionnaire for quality of life, Nurick scale for myelopathy and radiological parameters (segmental Cobb angle and cervical lordosis) were collected before surgery, at 6 weeks postoperatively and follow-up. **Results**: Seventeen patients met inclusion criteria. Mean age was 60.9 ± 7.6 years and mean follow-up was 12.9 ± 4.0 months. The NDI (55.4 ± 11.7 to 25.1 ± 5.4, *p* < 0.001) scores and the EORTC QLQ-C30 global health/QoL significantly improved postoperatively and at the last follow-up. The segmental Cobb angle (10.7° ± 5.6 to 3.1° ± 2.2, *p* < 0.001) and cervical lordosis (0.9° ± 6.7 to −6.2 ± 7.8, *p* = 0.002) significantly improved postoperatively. Only one minor complication (5.9%) was recorded. **Conclusions**: Carbon/PEEK implants represent a safe alternative to commonly used titanium ones and should be considered in cervical spinal metastases management due to their lower artifacts in postoperative imaging and radiation planning. Further larger comparative and cost-effectiveness studies are needed to confirm these results.

## 1. Introduction

Spinal metastases are the most common type of malignant lesions of the spine and approximately 5 to 10% of patients with systemic malignancy are affected [1,2]. The cervical spine is the least often involved by spinal metastases (10%), followed by the lumbar spine (20%), and the thoracic spine (70%) [3]. 

Spinal metastases are often diagnosed after providing neurological symptoms or painful conditions. Surgery may be palliative, rather than curative, aimed to improve their quality of life (QoL) [4,5,6]. For these reasons, minimally invasive surgery (MIS) techniques have become increasingly popular in spinal metastatic patients [7,8,9,10,11,12]. 

Anterior cervical corpectomy and plating is a well-recognized technique for the management of cervical spinal metastasis. Different reconstruction techniques of anterior vertebral body after corpectomy are proposed nowadays [3,12,13,14,15]. However, metal implants determine imaging artifacts and relevant diffraction and scattering effect during radiotherapy [16,17]. Therefore, alternative instrumentation materials with similar biomechanical characteristics along with lower effects on imaging and radiosurgery planning theoretically should be preferred in oncological patients [18,19]. Carbon-PEEK implants for thoracolumbar spinal procedures have been already reported as effective in spine tumors treatments, addressing the aforementioned issues [20,21,22,23]. 

This study aimed to firstly report the preliminary results from a cohort of patients with cervical spinal metastasis treated with anterior corpectomy and plating with Carbon-PEEK instrumentation and to evaluate the surgical, clinical and radiological outcomes.

## 2. Materials and Methods

### 2.1. Study Design and Guidelines

This is an observational study conducted in a single institution. The Strengthening the Reporting of Observational Studies in Epidemiology (STROBE) statement—checklist for cohort studies was used to define the study design. 

According to the study design and the non-modification of the standard of care, the IRB or ethical committee approval was not required. All the patients expressed written consent to the surgical procedure after appropriate information. Data reported have been completely anonymized. Therefore, this study is perfectly consistent, in any of its aspect, with WMA Helsinki Declaration of Human Rights.

### 2.2. Patient Population

Patients with cervical spinal metastasis who underwent anterior corpectomy and plating with carbon/PEEK instrumentation between March 2017 and September 2020 at our institution were prospectively considered for eligibility in the present investigation. A multidisciplinary team including neurosurgeons, oncologists and radiotherapists validated these inclusion criteria: symptomatic cervical spinal metastasis (neck pain non-responder to conservative medical treatments and/or tumor-related neurological symptoms); revised Tokuhashi score (TS) > 8 (intermediate and good prognostic group) [24]; spinal potential or confirmed instability (Spine Instability Neoplastic Score (SINS) ≥ 7) [25,26,27]. Exclusion criteria were: posterior involvement that requires a posterior approach; 2 or more contiguous affected vertebrae, grade 5 of Nurick scale [28].

### 2.3. Surgical Technique

A standard surgical technique using the Smith–Robinson approach was performed for anterior cervical corpectomy and plating. Cervical vertebral body replacement was made using a carbon/PEEK mesh cage with ostaPek^®^ composite (Trabis^®^, coLigne, Zurich, Switzerland) and a non-magnetic BlackArmor^®^ Carbon/PEEK anterior cervical plate (Icotec AG, Altstätten, Switzerland). Tantalum radiopaque markers indicate the anterior and posterior border of the mesh for checking its position with standard C-arm fluoroscopy. Moreover, these markers indicate the tip and the head of the screw, as well in Carbon-PEEK, for plate fixation and allows to track the progression of the screw through the vertebral body. A topic hemostatic agent and a drainage was left in the surgical site for 24 h in all patients.

### 2.4. Clinical and Radiological Outcomes

Demographical and clinical data were collected. The Neck Disability Index (NDI) questionnaire for neck pain and disability, European Organization for Research and Treatment of Cancer Quality of Life Questionnaire Version 3.0 (EORTC QLQ-C30 v. 3.0) for health and quality of life, and Nurick scale were collected before surgery and at 6 weeks and last follow-up (FU) visits. Radiological parameters evaluated were: segmental Cobb angle (angle between lines of superior endplate of the superior vertebra and the inferior endplate of the inferior vertebra of the pathological vertebra) and cervical lordosis (angle between lines of C2 and C7 inferior endplate). Preoperative, immediately postoperative and last FU X-rays, CT and MRI were also evaluated. The presence of mesh cage and plate dislocation, screw pullout or screw insertion angle changes and peri-implant loosening were considered as criteria for implant failure.

### 2.5. Statistical Analysis

Statistical comparison of continuous variables was performed by Student’s t-test. Statistical comparison of categorical variables was performed by chi-square statistic using Fisher’s exact test (2-sided). Differences were considered significant at *p* < 0.05. Statistical analyses were conducted using StatView version 5 software (SAS Institute Inc.).

## 3. Results

### 3.1. Demographical and Surgical Data

Seventeen patients underwent anterior corpectomy and plating with Carbon-PEEK instrumentation for cervical spine metastasis at our neurosurgical department in the investigation period and were included in the present investigation. 

The mean age of included patients was 60.9 ± 7.6 years (range, 48–74), and the M:F ratio was 0.89 (8M/9F). The mean follow-up was 12.9 ± 4.0 months (6–18). The primary tumor diagnosis was breast cancer in 6 (35.3%) patients, prostate adenocarcinoma in 5 (29.4%), lung adenocarcinoma in 3 (17.6%), kidney clear cell carcinoma in 2 (11.8%) and colon adenocarcinoma in 1 (5.9%). All patients reported mechanical neck pain non-responder to medical treatment, and Nurick scale was grade 0 in 4 (23.5%), grade 1 in 7 (41.2%), grade 2 in 3 (17.6%), grade 3 in 2 (11.8%) and grade 4 in 1 (5.9%) patient. The most common co-morbidity was cardiovascular diseases (64.7%), followed by diabetes mellitus (47.1%), obesity (23.5%) and respiratory disorders (17.6%). Four (23.5%) patients were smokers. Three patients were in ASA Class II (17.6%), 12 (70.6%) in class III and 2 (11.8%) in class IV. The pathological involved vertebra was C3 in 1 (5.9%) case, C4 in 8 (47.1%), C5 in 5 (29.4%), C6 in 2 (11.8%) and C7 in 1 (5.9%). The mean SINS was 10 (8–13). The mean TS was 10 (9–13). The mean length of surgery was 88.3 ± 28.5 min (60–180), mean estimated blood loss was 106.3 ± 39.8 mL (70–210), mean length of hospitalization was 3 (2–7) days, and every patient was mobilized within 24 h after surgery. No intraoperative complications were reported. All patients underwent radiation therapy within 3 weeks after surgery. The radiotherapy and chemotherapy varied based on primary tumor histology. Demographical and operative characteristics are summarized in Table 1 and Table 2.

### 3.2. Clinical and Radiological Outcomes

Mean NDI score improved from 54.4 ± 12.2 to 24.2 ± 3.8 at last FU (*p* < 0.001). EORTC QLQ-C30 global health/QoL improved from 17.9 ± 4.1 to 29.4 ± 5.1 (*p* < 0.001). The mean functional scale score changed from 53.5 ± 8.5 to 69.3 ± 7.7 at last FU (*p* < 0.001), and mean symptomatic scale score from 33.8 ± 5.4 to 18.5 ± 2.4 (*p* < 0.001). Nurick score improved in 11 (64.7%) patients, remained unchanged in 6 (35.3%) and none of the patients had a deterioration of neurological symptoms during follow-up. 

Mean segmental Cobb angle (10.7° ± 5.6 to 3.1° ± 2.2, *p* < 0.001) and mean cervical lordosis (0.9° ± 6.7 to −6.2 ± 7.8, *p* = 0.002) significantly improved after surgery.

**Figure 1:**
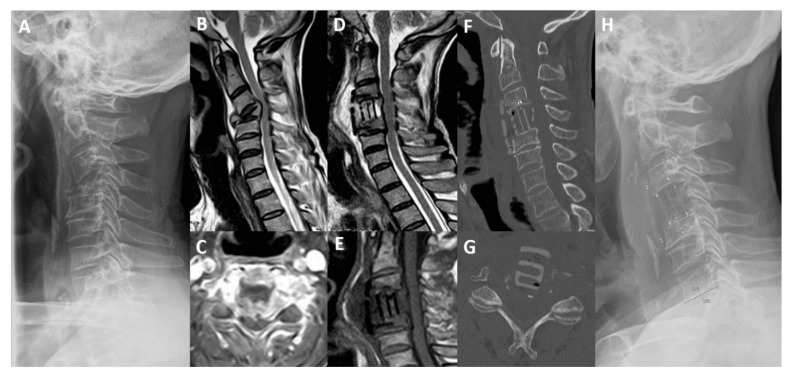
Magnetic resonance imaging (MRI), computed tomography (CT) and standing lateral radiograph of cervical spine. (**A**) Preoperative standing cervical lateral X-ray. (**B**) T2-weighted sagittal MRI. (**C**) T1-weighted with gadolinium axial MRI. (**D**) Post-operative T2-weighted sagittal MRI. (**E**) Post-operative T1-weighted sagittal MRI. (**F**) Post-operative (3 months) sagittal CT scan. (**G**) Post-operative (3 months) axial CT scan. (**H**) Post-operative standing cervical lateral X-Ray, showing a pathological fracture of C4 vertebra in a 59-year-old man with lung cancer and intractable neck pain and myelopathy. He underwent anterior C4 corpectomy and plate fixation with carbon-PEEK implants with resolution of pain and improvement of Nurick scale from grade 3 to 0. (TS = 9; SINS = 13).

Two patients died during follow-up for the evolution of primary cancer (7 and 10 months). Clinical and radiological outcomes are summarized in Table 3 and Table 4. An illustrative case is presented in Figure 1.

### 3.3. Complications and Reoperation Rate

No implant failures were reported. No major complications and one (5.9%) minor complication were recorded: one patient experienced a postoperative dysphagia that spontaneously recovered within 2 weeks. No patient required a reoperation during the follow-up.

## 4. Discussion

Surgery for spinal metastases is often considered as part of palliative care, eventually targeting to improve quality of life providing pain relief, restoring spinal stability and reducing the likelihood of further spinal cord compression [29,30]. The cervical spine is involved only in 8% to 20% of patients with vertebral metastatic cancer and the subaxial cervical spine is the most involved region [3]. Surgical treatment and techniques are influenced by the region of cervical spine affected. The anterior approach is the most common used to treat subaxial cervical metastases and combined anterior-posterior approaches could be considered in cases of multilevel and/or with circumferential involvement [14]. 

Anterior corpectomy and plating is usually preferred and different techniques for vertebral body reconstruction are proposed [13,14,15,31]. Additionally, combination of surgery and radiotherapy has been for a long time accepted as a gold standard treatment for spinal metastases management [32,33,34]. Titanium implants have been utilized in most cases over the last decades [35,36,37]. However, they determine artifacts on MRI and CT imaging, severely affecting post-operative imaging interpretation, in terms of accuracy and residual tumor visualization, and radiotherapy planning, in terms of precision and dose calculation [17]. 

The level of dosimetric effect of metal artifacts in radiation therapy depends on treatment modality (photon beam vs. proton beam). Different techniques can lead to reduction of the dosimetric error; however, further improvement is necessary. The effect can be more significant in particle therapy (PT), since currently available solutions are not sufficient to achieve the required dosimetric accuracy level [19]. Different studies have demonstrated that carbon-PEEK screws, compared with other materials, could favorably influence treatment efficiency and decrease possible over- and underdose of adjacent tissue with potential clinical advantages in the treatment of neoplastic disease [17,18]. Carbon-PEEK implant-related advantages in spinal instrumentations for spine tumors have been already reported [20,21,22,23]. 

In our series there was a significant improvement of pain and quality of life in all patients. Patient self-reported measures as NDI improved in all patients (54.4 ± 12.2 to 24.2 ± 3.8) from severe to mild/moderate disability in all patients. The EORTC QLQ-C30 quality of life (17.9 ± 4.1 to 29.4 ± 5.1), functional scale (53.5 ± 8.5 to 69.3 ± 7.7) and symptomatic scale (33.8 ± 5.4 to 18.5 ± 2.4) improved significantly after surgery. Moreover, the Nurick scale improved in 11 patients (64.7%) and there were no deteriorating symptoms during follow-up. All patients began radiotherapy and chemotherapy within 3 weeks after surgery. These results are consistent with other studies in the literature confirming that surgery improve the prognosis and quality of life of these patients [3,5,29,30].

This is the first prospective clinical investigation on Carbon-PEEK implants in cervical spinal metastases, evaluating only the biomechanical properties, surgical, clinical and radiological outcomes. The results of the present study confirmed the good results of the use of carbon/PEEK mesh cages in degenerative conditions [38,39]. However, there are no studies evaluating a Carbon-PEEK anterior cervical plate. Our results, compared to the titanium instrumentation in the literature [15,35,37], seem to suggest that Carbon-PEEK implants can provide similar surgical, clinical and radiological outcomes. On the other hand, MRI and CT scan images seem to be less affected by implant-related artifacts, thus resulting in a remarkable advantage of Carbon-PEEK for follow-up evaluations to detect early recurrence and/or progression. However, we did not observe any recurrence in this case series and it could be as a result of the short follow-up. Carbon-PEEK implant are more expensive than titanium ones, resulting in a final cost higher by as much as 30%.

In terms of system-specific technical difficulties, Carbon-PEEK implants are not visualized using fluoroscopes and are properly embedded with radiopaque marks. However, in our case series, there were no implant-related surgical complications or systems failure during follow-up. Furthermore, complication (5.9%) and reoperation rate (0%) are similar to those reported in the literature and commonly observed with titanium implants [40,41,42,43].

### Limitations

There are some limitations to be disclosed. The patient sample is relatively small and some complications and effects could have been consequently unrecognized. The follow-up duration was relatively short, although it was on an oncological case series. Moreover, a cost-effectiveness study is necessary to evaluate the additional cost of these implants compared to titanium ones. Finally, this study investigated only the feasibility and safety of the implants and the advantages of radiation planning and the theorical impact on survival were not considered and will be studied in further investigations. 

## 5. Conclusions

This investigation concludes that Carbon/PEEK implants for anterior cervical surgery reconstruction represent a feasible and safe alternative to commonly used titanium ones, reporting similar surgical and clinical outcomes, and should be considered in cervical spinal metastases management due to their lower artifacts in postoperative imaging and radiation planning. Further biomechanical investigations and comparative clinical trials would better clarify the role of carbon instrumentations and any advantage compared to standard titanium implants, especially related to radiation planning and its impact on survival.

## Figures and Tables

**Table 1:** Patient characteristics.

**Total number. of patients**	17
**Mean age ± SD, yrs (range)** **Mean Follow-up ± SD, mos (range)**	60.9 ± 7.6 (48–74) 12.9 ± 4.0 (6–18)
**Sex**	
Female Male	9 (52.9%) 8 (47.1%)
**ASA Classification**	
I II III IV V	0 3 (17.6%) 12 (70.6%) 2 (11.8%) 0
**Nurick Scale**	
0 1 2 3 4 5	4 (23.5%) 7 (41.2%) 3 (17.6%) 2 (11.8%) 1 (5.9%) 0
**Comorbidity**	
Cardiovascular diseases Diabetes Mellitus Obesity Respiratory diseases Smokers	11 (64.7%) 8 (47.1%) 4 (23.5%) 3 (17.6%) 4 (23.5%)
**Primary tumor**	
Lung Kidney Colon Prostate Breast	3 (17.6%) 2 (11.8%) 1 (5.9%) 5 (29.4%) 6 (35.3%)

**Table 2:** Operative characteristics.

	Nr. (%)
**Tumor Level**	
C3 C4 C5 C6 C7	1 (5.9%) 8 (47.1%) 5 (29.4%) 2 (11.8%) 1 (5.9%)
**Complications**	
Major Minor	0 1 (5.9%)
**Spinal Instability Neoplastic Score (range)**	10 (8–13)
**Revised Tokuhashi score (range)**	10 (9–13)
**Mean length of surgery ± SD, mins (range)**	88.3 ± 28.5 (60–180)
**Mean length of hospital stay, days (range)**	3 (2–7)
**Mean time of postoperative mobilization, days (range)**	1 (1–4)
**Estimated blood loss ± SD, ml (range)**	106.3 ± 39.8 (70–210)

Nr.: number

**Table 3:** Clinical outcomes.

	MEAN ± SD
**Neck Disability Index (NDI)**	
Preoperative	54.4 ± 12.2
Postoperative (6 weeks)	25.3 ± 4.3
Last follow-up visit	24.2 ± 3.8
*p*-value (pre vs. follow-up)	**<0.001**
**EORTC QLQ-C30**	
Quality of Life/Global Health *	
Preoperative	17.6 ± 4.1
Postoperative (6 weeks)	29.4 ± 5.1
Last follow-up visit	30.8 ± 4.1
*p*-value (pre vs. follow-up)	**<0.001**
Functional Scales *	
Preoperative	53.5 ± 8.5
Postoperative (6 weeks)	70.4 ± 7.4
Last follow-up visit	69.3 ± 7.7
*p*-value (pre vs. follow-up)	**<0.001**
**Symptomatic Scales ^§^**	
Preoperative	33.8 ± 5.4
Postoperative (6 weeks)	18.1 ± 2.7
Last follow-up visit	18.5 ± 2.4
*p*-value (pre vs. follow-up)	**<0.001**
**Nurick Scale**	**(n./%)**
Improved Unchanged Deteriorated	11 (64.7%) 6 (35.3%) 0

*: For QOL and functional scales, scores range from 0 to 100, and highest scores represent better quality of life; ^**§**^: For symptomatic scales, scores range from 0 to 100 and highest scores represent worst symptoms. Values in bold indicate statistically significant results.

**Table 4:** Radiological outcomes.

	MEAN ± SD
**Segmental Cobb angle°**	
Preoperative	10.7 ± 5.6
Postoperative (6 weeks)	2.7 ± 2.0
Last follow-up visit	3.1 ± 2.2
*p*-value (pre vs. follow-up)	**<0.001**
**Cervical lordosis°**	
Preoperative	0.9 ± 6.7
Postoperative (6 weeks)	−6.9 ± 8.1
Last follow-up visit	−6.2 ± 7.8
*p*-value (pre vs. follow-up)	**0.002**

° indicate that measure (grades); Values in bold indicate statistically significant results.

## Data Availability

Not applicable.
